# Multi‐functional microbial fuel cells for power, treatment and electro‐osmotic purification of urine

**DOI:** 10.1002/jctb.5792

**Published:** 2018-09-26

**Authors:** Iwona Gajda, John Greenman, Carlo Santoro, Alexey Serov, Plamen Atanassov, Chris Melhuish, Ioannis A Ieropoulos

**Affiliations:** ^1^ Bristol BioEnergy Centre, Bristol Robotics Laboratory Department of Engineering Design and Mathematics, University of the West of England Bristol UK; ^2^ Department of Applied Sciences University of the West of England Bristol UK; ^3^ Center for Micro‐Engineered Materials (CMEM), Department of Chemical and Biological Engineering University of New Mexico Albuquerque NM USA

**Keywords:** microbial fuel cell, urine, terracotta membrane, electro‐osmosis, Fe‐NCB catalyst, miniaturisation

## Abstract

**BACKGROUND:**

In this work, a small‐scale ceramic microbial fuel cell (MFC) with a novel type of metal–carbon‐derived electrocatalyst containing iron and nicarbazin (Fe‐NCB) was developed, to enhance electricity generation from neat human urine. Substrate oxidation at the anode provides energy for the separation of ions and recovery from urine without any chemical or external power additions.

**RESULTS:**

The catalyst was shown to be effective in clear electrolyte synthesis of high pH, compared with a range of carbon‐based metal‐free materials. Polarisation curves of tested MFCs showed up to 53% improvement (44.8 W m^−3^) in performance with the use of Fe‐NCB catalyst.

Catholyte production rate and pH directly increased with power performance while the conductivity decreased showing visually clear extracted liquid in the best‐performing MFCs.

**CONCLUSIONS:**

Iron based catalyst Fe‐NCB was shown to be a suitable electrocatalyst for the air‐breathing cathode, improving power production from urine‐fed MFCs. The results suggest electrochemical treatment through electro‐osmotic drag while the electricity is produced and not consumed. Electro‐osmotic production of clear catholyte is shown to extract water from urine against osmotic pressure. Recovering valuable resources from urine would help to transform energy intensive treatments to resource production, and will create opportunities for new technology development. © 2018 The Authors. *Journal of Chemical Technology & Biotechnology* published by John Wiley & Sons Ltd on behalf of Society of Chemical Industry.

## INTRODUCTION

Water is a naturally circulating resource that is constantly recharged however climate change, increasing population and associated shifts in the water cycle vastly complicate the challenge of sustaining freshwater supplies for the future. The access to water in quantity and quality is one of the key issues affecting human development and is a main driver for circular economy growth. A ‘circular economy’ would turn goods that are at the end of their service life into resources for others, closing loops in industrial ecosystems and minimising waste. Bioelectrochemical systems such as microbial fuel cells (MFCs) have the capacity to add value to otherwise useless organic waste streams and to generate off‐grid electricity and may be one of the most interesting examples of renewable energy sources.^1^, ^2^, ^3^ In the MFC, the electric field is created by the oxidising bacterial consortia in the anaerobic anode utilising organic substrates from the feedstock (wastewater, urine) directly into electric current and protons.^4^, ^5^, ^6^ While the electrons travel through the circuit, the corresponding protons migrate to the cathodic compartment through an ion‐exchange membrane to reduce oxygen. One of the bottlenecks of MFC performance is the oxygen reduction reaction (ORR) occurring at the cathode. Cathodic overpotentials are one of the main contributors to overall performance losses in MFCs^7^ therefore the cathodic conditions need to be optimised in terms of the electrode materials with catalytic activity. Platinum is widely used as a catalyst for air cathodes in laboratory systems, but at the same time it is infeasible for large‐scale developments due to its high cost and high susceptibility to poisoning mainly due to anions and deactivation of its active sites.^8^ So far, the cathodic performance has been hindered by cathode fouling due to clogging,^9^ salt deposits^10^, ^11^ and biofilm growth.^12^ Even most recent studies report that this remains an issue^11^, ^13^ suggesting the existence of both external and internal fouling.^9^ Therefore prolonged lifetime of the cathodes as well as the other elements of the system is critical for economical applications of MFCs.

Developing an inexpensive electrocatalyst with a porous structure, large surface area, and abundant active sites for efficient cathodic oxygen reduction reaction (ORR) in MFC is still highly desirable.^14^ Especially considering cost‐effective activated carbon (AC), its catalytic activity to ORR can be attributed to the additional sites provided by the micro‐ and mesoporous structure within the high surface area which is an important factor for its excellent performance in circum‐neutral operating electrolyte.^15^, ^16^ Activated carbon (AC) has a long history of applications in environmental technology as an adsorbent of pollutants for the purification of air and water. Moreover, it is widely used in MFC technology as inexpensive gas diffusion cathode,^17^ as a robust candidate suitable for the stacking of a number of MFCs in practical demonstrations.^18^

In general, the most efficient catalysts undergo a direct 4‐electron transfer mechanism process and the least efficient through a 2‐electron mechanism (peroxide pathway)^19^ and recently it has been shown that Fe‐based catalysts follow a 2 × 2 e‐transfer mechanism.^20^ Highly active and durable iron–nicarbazin derived electrocatalyst (Fe‐NCB) was originally developed for future automotive applications synthesised from iron nitrate and nicarbazin (NCB) as iron salt and nitrogen rich organic precursor.^21^ Fe‐NCB was successfully used in a recent MFC study^22^ and was shown to be a promising ORR catalyst.

The feasibility and stability of iron‐based catalyst in long‐term MFC operation has previously been demonstrated,^23^ however in preparation for real field trials and applications, this catalyst needs to be tested in terms of the suitability for urine treatment since it has been shown that ceramic MFCs are promising for catholyte extraction and nutrient recovery. Due to a high concentration of nutrients, urine can be considered as a valuable resource for N, P and K^24^, ^25^, ^26^ and energy recovery,^27^therefore suitable technology advancements are needed to allow nutrients to be separated from urine close to the source.

The aim of the current study was to test a range of affordable and simple in preparation carbonaceous cathode materials for urine fed MFCs. Fe‐nicarbazin derived catalyst was therefore incorporated into air‐breathing cathodes in order to be tested as a simple, tri‐generative system (i) to improve electricity levels in order to upscale the system for practical applications, (ii) to efficiently treat waste (neat human urine) in the anode, and (iii) to obtain electrochemically treated (transparent) catholyte as a function of system performance. Optimising the power output in a simple manner will be an effective and novel approach for improving the energy recovery, optimising the anode chamber as the feed side and the engine of the electro‐osmotic process and the cathode chamber as the permeate side for the self‐driven filtration and recovery of water from urine. The novelty of this process is in the simplicity of the operation, chemical‐free and self‐sustaining recovery. It is vital to explore commercially applicable environmental catalysts in order to apply the technology in the real world.

This is the first time that the MFC has been presented not just as an organic waste consuming and simultaneous electricity producer but also as an electro‐osmotic filtration device for urine cleaning. The phenomenon of catholyte production and accumulation is presented in the context of an important electricity‐driven active‐filtration mechanism in which the MFC is a trigenerative system for the production of electricity, clean catholyte and COD removal. Taking advantage of the high pH of the extract formed in the cathode, ammonia stripping or struvite precipitation for the recovery of nutrients and water from urine can be achieved.

## MATERIALS AND METHODS

### Catalyst preparation

Iron–nicarbazin derived catalyst used in this experiment was synthesised using a Sacrificial Support Method (SSM). The optimised procedure was previously described.^28^ Nicarbazin (NCB), silica and iron nitrate were mixed together. Nicarbazin, as nitrogen‐rich organic precursor, was initially dispersed in water and then mixed with two different types of silica: (i) OX possessing a surface area of roughly 50 m^2^ g^−1^; (ii) in‐house synthesised monodispersed silica possessing a surface area of roughly 10 m^2^ g^−1^ with a 50 nm particle size. During the synthesis, silica was used as templating agent. Iron nitrate (Fe(NO_3_)_3_*9H_2_O) was then added to the suspension of nicarbazin and silica and then ultrasonicated to obtain fine dispersion. The mixture was subjected to heat treatment at T = 85°C in normal atmosphere to allow water evaporation and then was ground using a mortar and a pestle. Having obtained a fine powder, the mixture was subjected to pyrolysis in an inert atmosphere (UHP N_2_ at a flow rate of 100 ccm). The temperature was increased at a rate of 75°C min^−1^ to 975°C and maintained at this temperature for 45 min. After this, the sample was cooled down to room temperature. Etching of the silica template followed the pyrolysis procedure. The fine black powder was in fact etched using diluted HF (25 wt%) for at least 1 day. HF was washed away through further dilution until the pH of the liquid reached neutrality. The powder was then subject to dry procedure in atmosphere at a temperature of 85°C for roughly half a day. The powder was then subject to an additional pyrolysis treatment in a reducing atmosphere (NH_3_ at a flow rate of 100 ccm) in order to reduce the graphitised carbon. The temperature was increased at a rate of 75°C min^−1^ to 975°C and maintained at that temperature for 30 min.

### Cathode electrodes preparation

**Figure 1:**
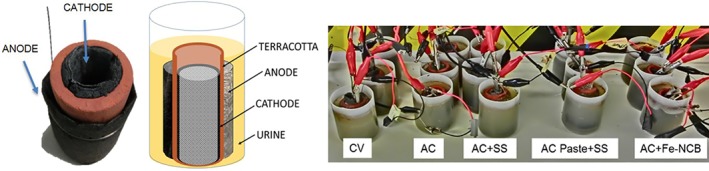
Ceramic MFC with an outer anode and inner cathode configuration and the experimental set up including the triplicates of each tested condition.

Five different carbon based cathode materials were prepared as shown in Table 1. CV type was made of carbon veil fibre (20 g m^−2^, PRF Composites, UK) with dimensions 200 × 280 mm which was folded, rolled‐up and placed inside the ceramic cylinder filling the whole available space inside. AC electrodes (used here as control) were prepared by mixing activated carbon powder (G Baldwin and Co, UK) with polytetrafluoroethylene (PTFE, 60% in H_2_0, Sigma Aldrich) (8:2) wt/wt ratio and deionised water into a smooth paste and applying it onto PTFE treated (30%) carbon veil matrix as described previously.^29^ The resultant cathode electrode material of 3 mm thickness was then cut to the required dimensions, 45 mm by 50 mm; the cathode was then placed inside the cylinder (Fig. 1). AC + SS cathodes were prepared adding a rectangular piece of 45 mm by 50 mm stainless steel (SS) mesh of 0.28 mm diameter (316 grade, Streme Ltd, UK) as a current collector to the control AC cathode. The mesh was placed after the AC was inserted and was in direct contact with the carbon veil backbone of the AC cathode. AC paste was constructed around the metal mesh itself, thereby avoiding the need for the carbon veil as supporting material without carbon veil matrix (AC paste+SS) to explore this type of modification. AC paste was first applied onto the inner walls of the cylinder and then a piece of stainless steel was added (45 mm by 50 mm) for current collection and air dried. Finally, AC + Fe‐NCB electrodes were prepared by mixing 2 mg cm^−2^ of the Fe‐NCB electrocatalyst into the activated carbon paste prepared in the same way as for the control AC.

**Table 1:** Cathode types tested in this work

Acronym	Type of cathode	Cathode construction
**CV**	Carbon veil	Carbon veil fibre folded and inserted into the cathode chamber
**AC**	Activated carbon	Activated carbon applied on PTFE treated carbon veil
**AC + SS**	Activated carbon with stainless steel mesh	Activated carbon applied on PTFE treated carbon veil with added stainless steel mesh for current collection
**AC paste + SS**	Activated carbon paste with stainless steel mesh	Activated carbon paste applied directly onto the inner side of the ceramic with added stainless steel mesh for current collection
**AC + Fe‐NCB**	Activated carbon with Fe‐NCB catalyst	Activated carbon with added Fe‐NCB catalyst applied on PTFE treated carbon veil

### MFC assembly and operation

MFCs were assembled using terracotta cylinders (Jain Scientific Suppliers, India) that were sealed at one end creating a hollow space inside (Fig. 1). The cylinder dimensions were 50 mm long, 22 mm inside and 30 mm outside diameter, with an internal volume of 10 mL. Anodes were made of 200 × 280 mm carbon veil fibre (20 g m^−2^, PRF Composites, UK) which was folded four times with a projected area of 45 cm^2^ and wrapped around the cylinder as shown in Fig. 1 using stainless steel wire for anode connection. All the anodes were prepared the same way in all experimental groups. The cathodes were placed in the inner chamber of the cylinder; the CV based cathode was folded and rolled inside with a stainless steel crocodile clip attached for current collection. After fabrication, the AC based cathodes were inserted into ceramic cylinders with the activated carbon layer exposed to the ceramic wall and the current collector exposed to the atmosphere using stainless steel crocodile clips as connectors to the external circuit and data acquisition hardware. The cathode geometric area in contact with the ceramic separator was 22.5 cm^2^. Assembled MFCs were then placed in plastic containers as shown in Fig. 1 where each reactor was seeded with a 45 mL concentrated mixed inoculum derived from activated sludge and urine mixed in equal amounts and used as inoculum. After that, the anolyte was emptied and refilled with fresh urine in batch mode. For the following feeding cycles, neat human urine was used as substrate for the MFCs. Urine was collected anonymously from healthy individuals, pooled together and stored in a collection tank (pH 8.9–9.4), at room temperature and used directly as the feedstock for the MFCs.

### Measurement and analysis

The MFC voltage was recorded every 3 min by a multi‐channel 24 ADC PicoLog Data Acquisition Unit, recording output in mV. Current (I) and power (P) were calculated using Ohm's law, where R is the known external resistor value and V is the recorded voltage value. The volumetric densities of power and current were calculated based on the liquid volume of the anode compartment. Polarisation experiments were performed on single MFC units by applying a range of resistance values from 30 kΩ down to 3 Ω using an automated variable resistor every 3 min during which time, data were recorded every 30 s.^30^

The physicochemical properties of urine and formed catholyte were analysed using a 8424 pH meter (Hanna Inst., UK) and Jenway 470 conductivity meter (Camlab, UK). The concentrations of chemical oxygen demand (COD) were analysed on filtered anolyte samples (0.45 μm, Millex, USA) using the potassium dichromate oxidation method (COD HR, Camlab, UK) and a MD 200 photometer (Lovibond, UK). Cation concentration was analysed using ion chromatography (IC 930 Compact IC Flex, Metrohm, UK) where the anolyte samples were taken before and during the MFC treatment.

## RESULTS AND DISCUSSION

### MFC power performance

**Figure 2:**
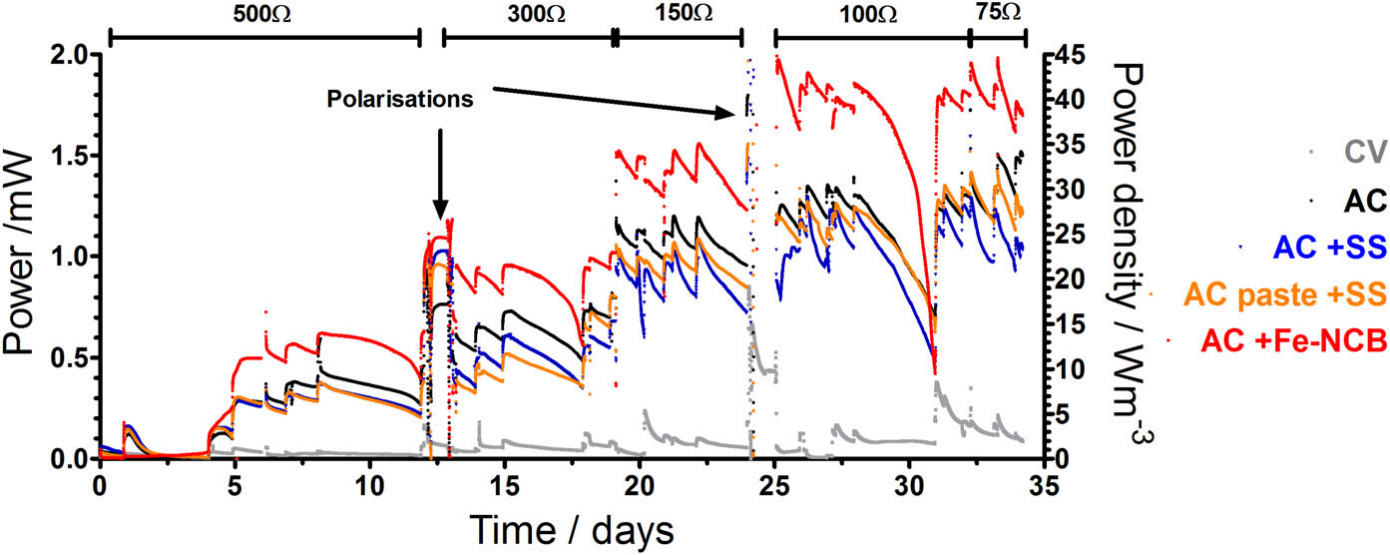
Temporal data showing the power performance over 35 days of the MFC operating in batch mode: the arrows indicate the polarisation experiments performed.

The performance was monitored over 35 days from the experiment start and the initial external resistance applied was 500 Ω; after further adjustment to 300 Ω and 150 Ω during the maturing stage whereas the optimal performance was achieved with 100 Ω and 75 Ω, as indicated in Fig. 2. Average performance under 100 Ω of the MFC with AC + Fe‐NCB electrode showed a power generation of 1.82 mW (1.99 mW maximum) while the AC reached 1.26 mW (1.51 mW maximum). The data indicate that the catalyst is performing 44% better than the AC control. Batch operation was undertaken to control and measure feedstock utilisation in terms of power production, COD removal and catholyte generation over time to understand the physicochemical changes occurring in the given portion of feedstock while producing current. Periodic feeding resulted in an increase in performance followed by a slow decay of power due to the depletion of easily available organics and nutrients that were being utilised in batch mode operation, which will be discussed further in the text. The heavier the external load used, the higher the performance achieved and the quicker the rate of substrate depletion, which can be seen as faster reduction of power.

The results demonstrate that the addition of stainless steel as a current collector both to control as well as embedded into the AC paste does not significantly affect the output however the addition of electroctive catalyst to the AC contol showed up to 44% improvement in power performance and superior performance was obtained throughout the duration of the experiment.

### Polarisation experiments

**Figure 3:**
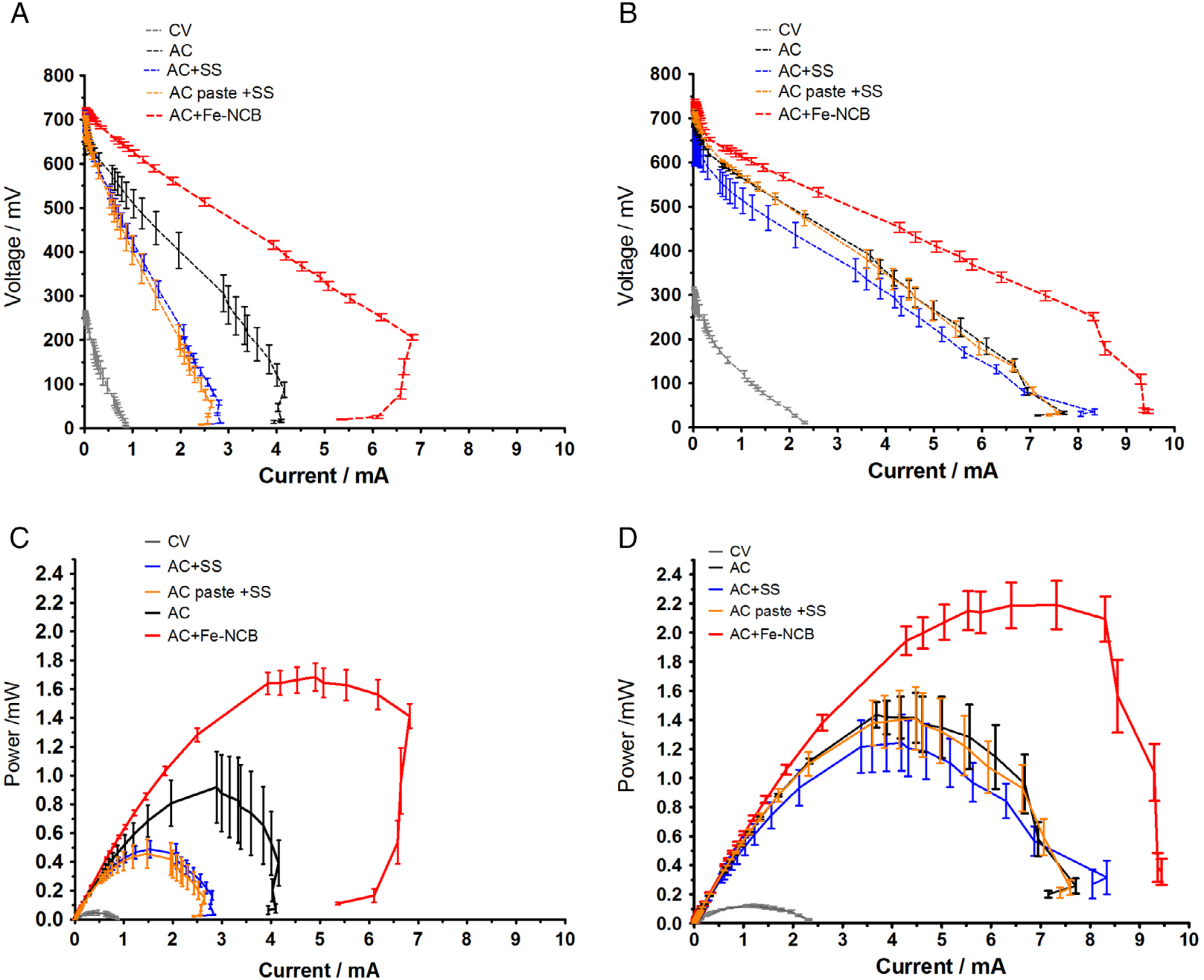
Polarisation values of all tested MFCs after 12 days of operation (A), and after 24 days (B). Power curves of all tested MFCs from maturing after 12 days of operation (C), and established MFCs after 24 days (D) (error bars represent SD of triplicated data).

The initial polarisation experiment was performed after 12 days of MFC operation to investigate if the MFC anodes had matured. Power curves (Fig. 3(C)) showed a maximum of 1.68 mW (33.6 Wm^−3^) for the AC + Fe‐NCB, 0.92 mW (18.4 Wm^−3^) for the AC, 0.49 mW (9.8 Wm^−3^) for AC + SS and 0.46 mW (9.2 Wm^−3^) for the AC paste + SS. CV achieved only 0.05 mW (1.0 Wm^−3^) which might be due to the absence of a catalytic layer in the form of activated carbon or any other catalyst, while the overshoot^31^ might suggest the anodes are not fully matured. Similar open circuit voltage (OCV) was measured for all AC based materials and a significantly lower value was recorded for CV. Following the results of this test the external load was adjusted to 300 Ω and 150 Ω due to power increase over time. A second polarisation was performed after stabilising at day 24. Figure 3(D) shows that the maximum power 2.19 mW (44.8 Wm^−3^) was achieved by the AC + Fe‐NCB, 1.43 mW (28.6 Wm^−3^) for the AC, 1.24 mW (24.8 Wm^−3^) for AC + SS and 1.41 mW (28.2 Wm^−3^) for the AC paste + SS. CV achieved only 0.12 mW (2.4 Wm^−3^). Data from the polarisation experiments show that Fe‐NCB improved power generation by 53%, which is consistent with the temporal data. The choice of Fe‐NCB as a catalyst appears to be a suitable option for enhancement of MFC performance. Clearly, this strategy offers more efficient extraction of the chemical energy locked in urine.

### Urine treatment

**Figure 4:**
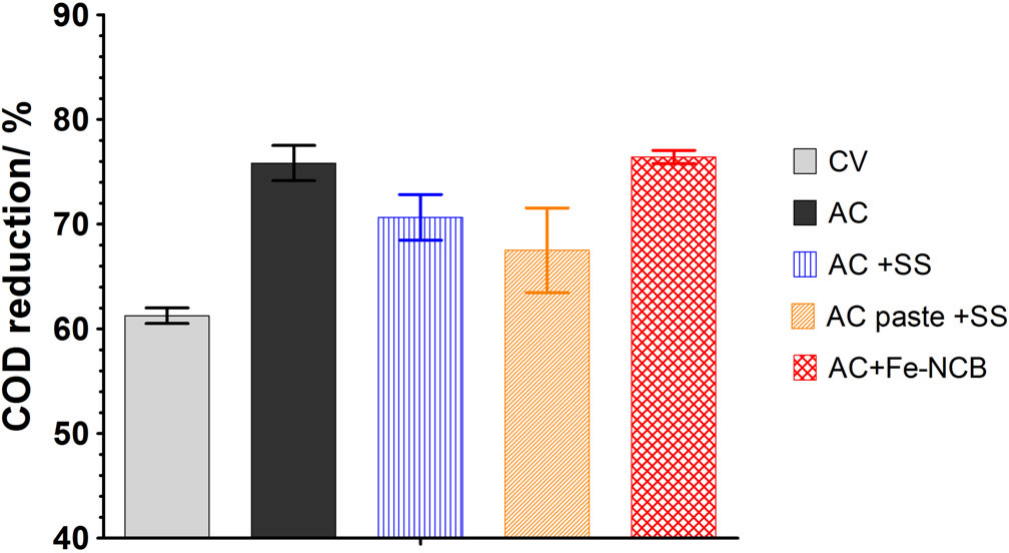
Urine COD reduction in tested MFCs. Measurements were taken after 8 days of MFC operation, and the data presented are the mean for n = 3.

MFC performance was also evaluated in terms of urine treatment capacity by measuring the chemical oxygen demand (COD) removal over time. For this purpose, the evolution of the COD in the anode chamber was checked. The COD removal trends (Fig. 4) show that both AC and AC + Fe‐NCB based systems are the most efficient in organic removal at the anode, which can be linked to their electrical performance, as the worst‐performing CV showed lowest COD reduction rate (Fig. 4).

**Figure 5:**
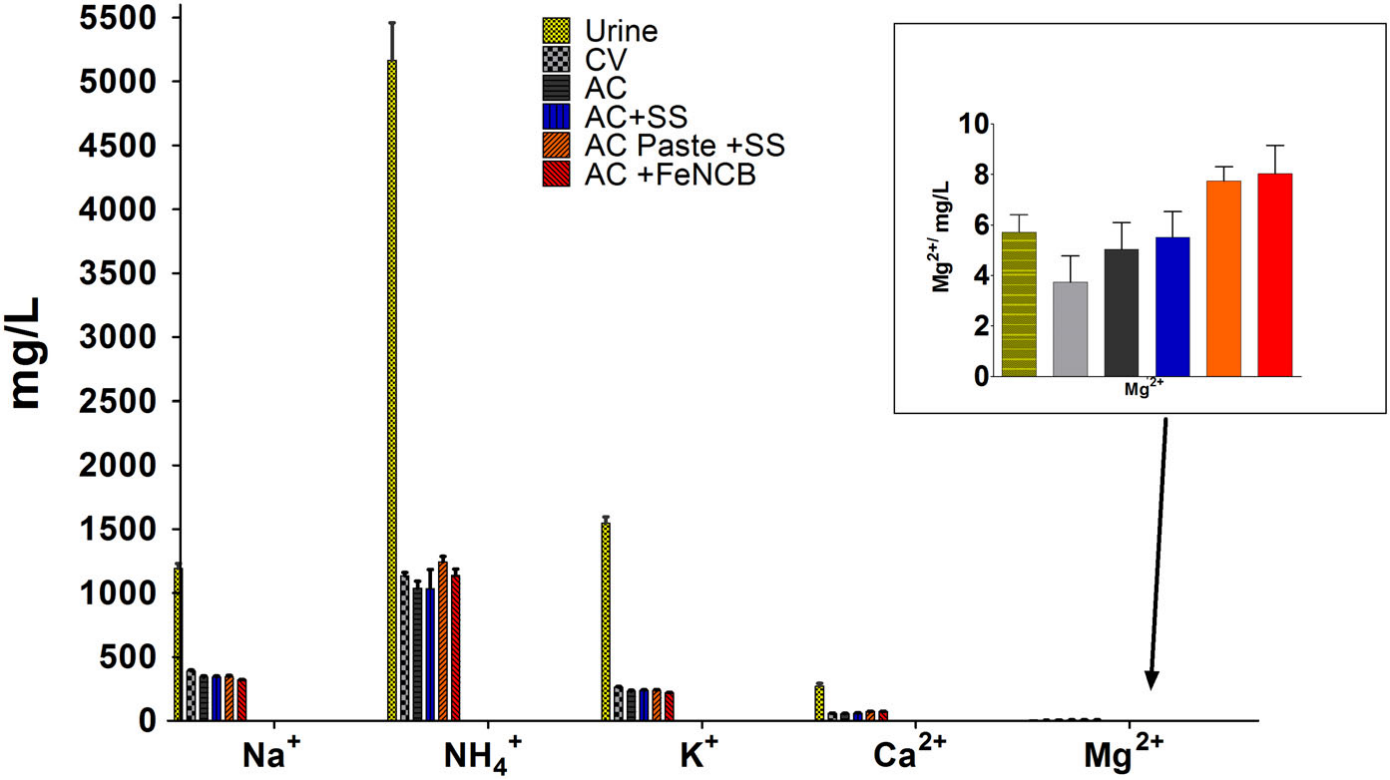
Concentration of cations in urine before treatment and after MFC operation measured in the anode in each experimental group.

The concentration of cations measured in the anolyte before and after treatment in Fig. 5, follows a similar trend, where the concentrations of Na^+^, K^+^, NH_4_^+^, Ca^2+^ ions show significant decrease. This might be attributed to the transport of the positive charge into the cathode. The ion present at the highest concentration was ammonium, which could potentially be volatilised into ammonia (NH_3_) in high pH solution in the cathode chamber.

### Catholyte extraction and electrochemical treatment

MFC operation results in continuous production of new diluent that was recovered as a catholyte liquid from the inner chamber of the MFC, which was initially empty. This was previously observed in similar ceramic MFCs treating wastewater enriched with sodium acetate.^29^ To further study this phenomenon in the current set‐up, the cathodes were emptied and monitored over 8 days to assess the quantity and quality of newly formed catholyte. In chemical fuel cells fed with hydrogen or methanol, the water distribution is determined by the interplay among water uptake by the membrane, electro‐osmotic drag from anode to cathode, water production at the cathode and back‐diffusion of water due to the activity gradients. Specifically, the electro‐osmotic drag consists in the transport of water molecules with the protons moving from the anode to the cathode and therefore it is directly correlated with the current produced by the fuel cell. Similarly, in operating MFCs, the increase in current/power generation enhances the transport of protons and other cation species flowing between the anode and the cathode, therefore more liquid is transported from the anodic to the cathodic chamber. Moreover, cathode alkalinisation occurs due to the production of OH‐ during the oxygen reduction reaction increasing the concentration of negative charges in the cathode. Consequently, for electroneutrality, cations move to the cathode.

**Figure 6:**
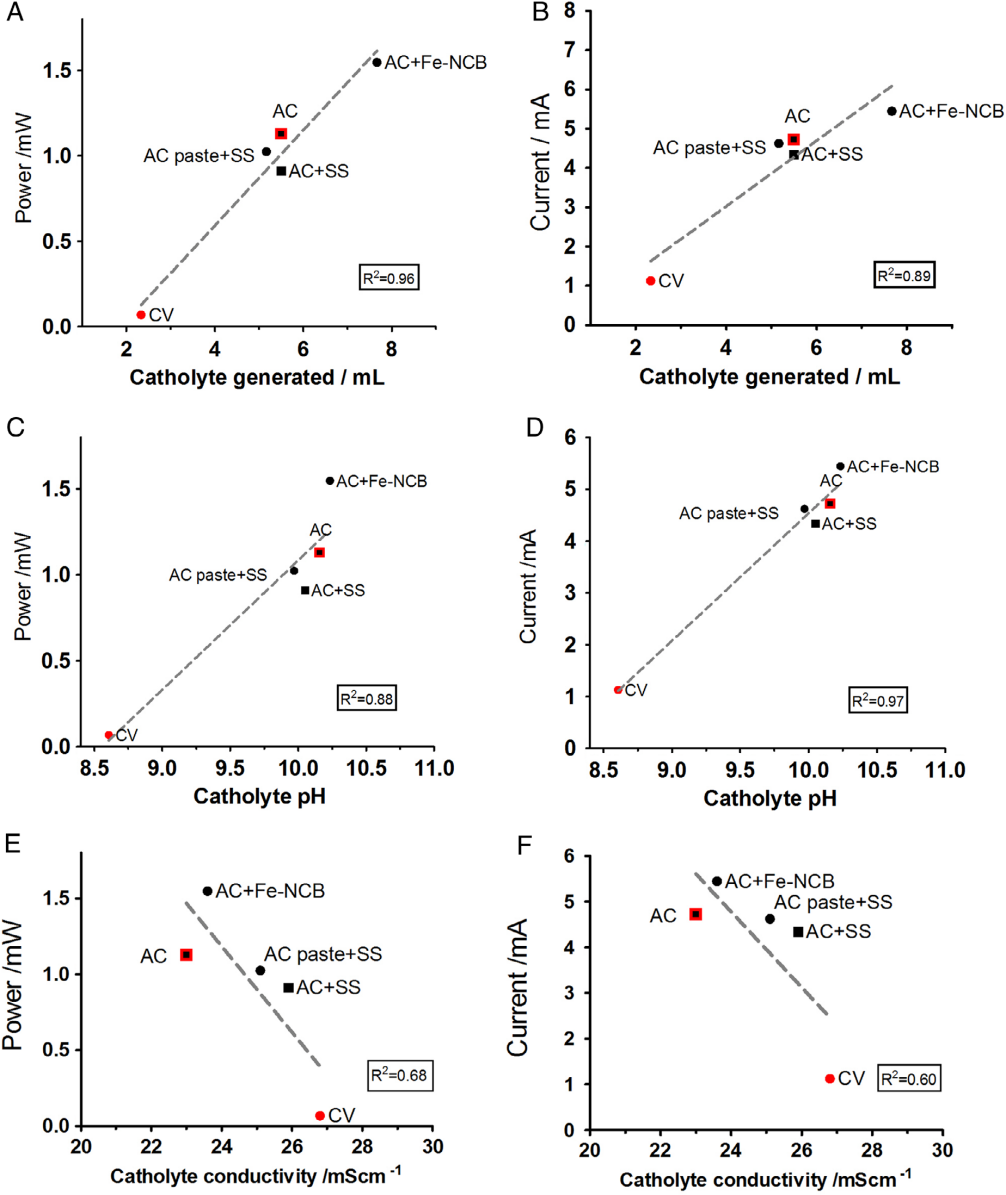
Power performance in relation to the catholyte volume (A), catholyte pH (C) and catholyte conductivity (E). Current generation in relation to the catholyte volume (B), catholyte pH (D) and catholyte conductivity (F).

A greater quantity of catholyte was collected from the best performing MFCs (Fig. 6(A,B)). The linear relationship between power/current output and the amount of accumulated catholyte as shown in Fig. 6(A,B) underlines the fact that the electro‐osmotic force is dominant as previously shown by Merino‐Jimenez *et al*. in a cylinder of similar thickness treating urine.^32^ Recent studies show MFC integration into a membrane separation process to purify the effluent,^33^ however, on the basis of electro‐osmosis and the production of catholyte it can be proposed to use the ceramic MFC to filter the anolyte through the electro‐osmotic force of the electricity produced without any external power addition. Electro‐osmosis in polymer electrolyte membranes leads to water management issues and cathode flooding,^34^ however here for the purpose of urine filtration it is particularly useful. While ceramic has been used in filtration technologies, it has never been studied as the electro‐osmotic filter for urine purification in a MFC. The filtration characteristics of ceramic membranes are affected by the physical, chemical and electrochemical properties and for the purpose of their integration within MFC systems they still need to be characterised however they are promising alternatives for urine purification.

It is interesting to observe that in this set‐up the catholyte conductivity diminishes with power/current (Fig. 6(E,F)) which is in line with previous work on urine operated systems at lab scale^32^ and in field trial‐systems.^18^ In general, it can be observed that in urine operated MFCs, the catholyte produced has lower ionic strength than the influent (raw urine) thanks to the higher rate of electro‐osmosis (due to higher power) dragging water molecules against the osmotic gradient thus producing more diluted catholyte with increased power. When ions are transported through an ion‐exchange membrane, they are accompanied by a hydration shell of water molecules, which is defined as electro‐osmotic drag.^35^, ^36^ Also, in addition to electro‐osmotic transport, osmotic water transport takes place and depends on the concentration difference between the concentrate and the diluate separated by the membrane. Improved efficiency of the power performance results in improved catholyte generation, pH splitting and ion separation. The pH splitting mechanism is observed in relation to MFC performance (Fig. 6(C,D)) and this can be explained by H^+^ production on the anode and OH^−^ production at the cathode.

**Figure 7:**
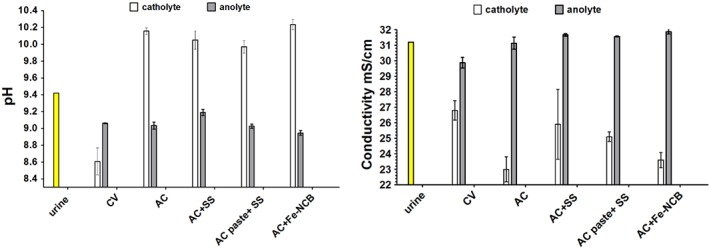
Physico‐chemical analysis of the anolyte and catholyte in tested MFCs in comparison with fresh influent (urine).

Figure 7 shows that the pH splitting force increases in those MFCs performing the best as the difference between anolyte and catholyte pH is the highest in AC + Fe‐NCB (1.3 units) and lowest in the underperforming CV (0.4 units). Ion splitting follows a similar trend whereby the Fe‐NCB and AC demonstrate the highest separation rate (>8 units) between the ionic content in the anode and the cathode after MFC treatment in comparison with the low‐performing CV (3 units). Therefore the catholyte formed is more diluted and transporting water molecules against osmotic gradient.

**Figure 8:**
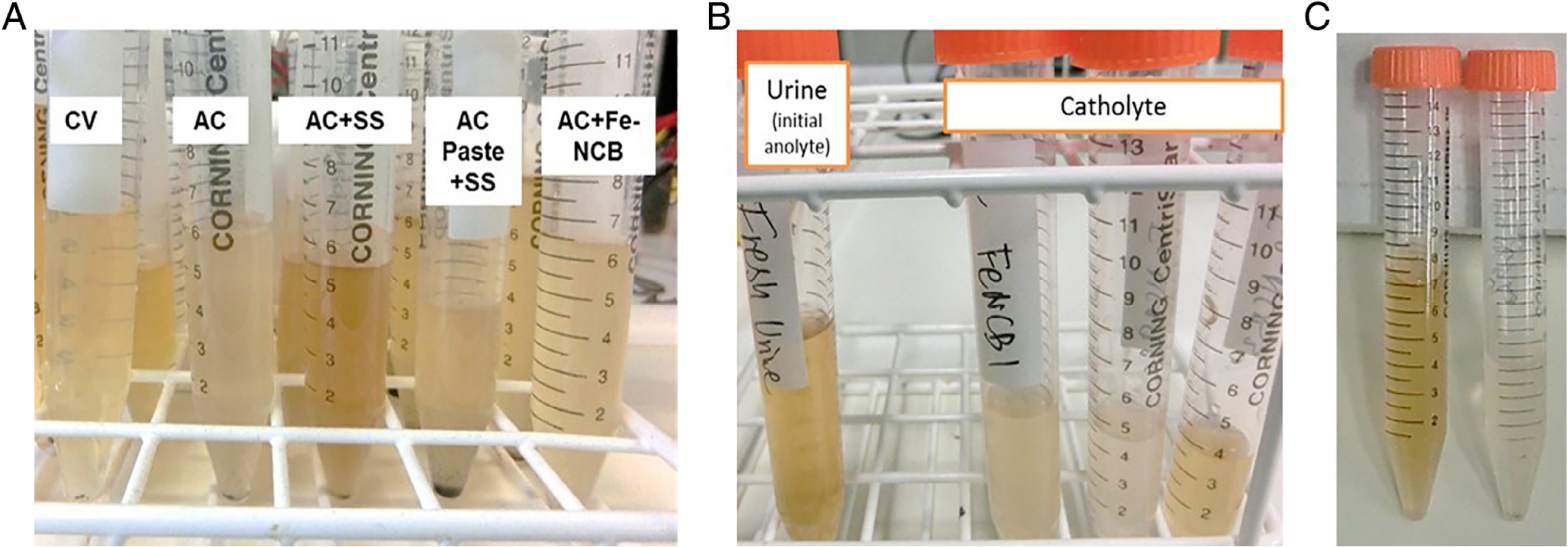
Visual evidence of catholyte bleaching: (A) catholyte collected from all tested MFC groups at a different point in time relative to the data depicted in Fig. 6, (B) catholyte samples from the AC+Fe‐NCB (MFCs 1, 2 and 3) show the discoloration (bleaching) of the catholyte in comparison with fresh urine, (C) fresh urine sample used as feedstock (anolyte) in comparison with catholyte from AC+Fe‐NCB catalyst.

Also, the catholyte collected from the AC + Fe‐NCB and AC MFCs is paler than the catholyte obtained from the other MFCs (Fig. 8(A)) showing that bleaching was most visible in the samples obtained from AC + Fe‐NCB cathodes (Fig. 8(B)) in comparison with fresh urine (Fig. 8(C)). This might suggest that the extract obtained is more diluted due to higher electro‐osmotic transport (higher current) as this is supported by the lower conductivity values (Fig. 6(E,F)) or through the improved activity of the ORR and concomitant production of reactive species. During the ORR, the production of hydrogen peroxide rapidly accelerates the production of •$O_{2}^{-}$ and •OH radicals.^19^ Therefore after efficient MFC operation, more purified and cleaner catholyte can be generated and extracted from the anolyte which in turn becomes more concentrated (Fig. 7). The specific transport needs to be studied further in order to better understand the process and to explore the formation of reactive oxygen species (ROS).

This feature is taking advantage of the design aiming for catholyte self‐accumulation in the inner chamber without the need for supplying external power or adding a drag solution as in osmotic MFC systems.^37^ In air‐breathing cathodes which are completely open to the air, the precipitation of transported ions occurs due to loss off humidity and the cathode drying out. These changes have been attributed to formation of salt deposits as well as the formation of cathode biofilm blocking proton transfer to the catalysts and reducing oxygen diffusion leading to decrease in power production.^38^ However, it has been shown that by appropriate cathode chamber modification limiting evaporation and not allowing the cathode electrode to dry out, therefore not letting salt deposits to form, the cathode could function as the catholyte producing chamber^39^, ^40^ even in long‐term operation of 1 year.^23^ This configuration allows the formed catholyte droplets to be self‐hydrating, washing the deposits off in order to prevent salt deposition and maintaining continuous production of catholyte. Moreover, a similar concept in the ceramic based MFCs led to the formation of antimicrobial, high pH catholyte protecting from biofouling.^41^ Formation of catholyte is strongly connected to the current induced ionic transport^35^, ^42^ which can lead to ammonia recovery and ammonia stripping.^43^

The separation process uses the advantage of current production by the MFC, which drives water and ions transport across the membrane; this is different from the energy intensive electrodialysis systems where the electricity is supplied externally. While all desalination technologies have high costs this might be one solution to pursue in order to diminish the salt content. Therefore, future experimental work should be directed towards developing electrodes capable of higher current densities in preparation for technology readiness for real‐life applications. Due to oxygen reduction reaction at the cathode and formation of OH^−^, ROS, hydrogen peroxide and other reactive chemical species,^19^, ^44^ the catholyte might have disinfecting capabilities similar to peroxide or bleach.^41^ Urine could then be passed through the system and simultaneously treated through biological (COD reduction) and electrochemical processes (catholyte extraction) within the MFC. The system benefits from the direct generation of power *in situ* to run the separation. This is particularly important for implementation in field trials of this technology^18^ where the application of catalyst would improve the output and the catholyte cleaning capabilities and to improve sanitation. Current production directly influences the water flux rate and ion migration, however, ion migration during nutrient recovery needs to be investigated further.

Compared with a traditional energy intensive treatment‐focused approach, this integrated process takes advantage of the electric current generated during wastewater treatment driving the anodic processes (COD treatment) and ion transport leading to catholyte extraction and purification. To enhance the output, this system should be studied further in order to optimize purification capabilities and implement it in practical applications. Similar ceramic units were configured into an MFCs stack and investigated with human urine in real experimental conditions during a recent field trial in rural Uganda showing a potential for future development of multi‐functional MFCs.^45^

## CONCLUSION

This work describes an efficient oxygen reduction catalyst for the microbial fuel cell. The power density achieved in this study with the utilisation of Fe‐based catalyst achieved higher power generation compared with AC cathodes. In contrast with acrylic MFCs, the ceramic MFCs offer simple operation, lower cost and can accomplish the recovery of valuable resources such as energy, water, and nutrients from urine.

The study confirmed that current generation was also a key factor to drive the water extraction, pH and ion splitting and showed the MFC to be an electro‐osmotic filtration system bleaching urine. Ion concentration, pH and rate of catholyte production relationships showed a linear dependence with power/current generation showing pH and ion splitting as well as water extraction that underlines the ability of MFC system to generate electric power and simultaneously clean human urine. Using the MFC as the platform technology, this configuration can also be beneficial for other bioelectrochemical systems in order to simplify the design for elemental recovery.
